# Histological Evaluation of Oral Soft Tissue Biopsy by Dual-Wavelength Diode Laser: An Ex Vivo Study

**DOI:** 10.3390/dj13060265

**Published:** 2025-06-13

**Authors:** Daniele Pergolini, Alessandro Del Vecchio, Mohamed Mohsen, Veronica Cerullo, Cinzia Angileri, Eduardo Troiani, Paolo Visca, Barbara Antoniani, Umberto Romeo, Gaspare Palaia

**Affiliations:** 1 Department of Oral and Maxillofacial Sciences, Sapienza University of Rome, 00161 Rome, Italy; daniele.pergolini@uniroma1.it (D.P.); alessandro.delvecchio@uniroma1.it (A.D.V.); veronicacerullo18@gmail.com (V.C.); angileri.1899997@studenti.uniroma1.it (C.A.); troiani.1831250@studenti.uniroma1.it (E.T.); gaspare.palaia@uniroma1.it (G.P.); 2 Department of Citology and Cellular Diagnostics, Regina Elena Institute, Via Elio Chianesi 53, 00144 Rome, Italy; visca@ifo.it (P.V.); barbara.antoniani@ifo.gov.it (B.A.)

**Keywords:** diode laser, ex vivo, histological evaluation, laser surgery, oral biopsy, thermal damage

## Abstract

**Background:** Diode lasers are valuable in oral surgery due to their excellent hemostasis, minimum post-operative pain, and minimally invasive procedures. A dual-wavelength diode laser in dentistry combines two distinct wavelengths, typically 450 nm and 808 nm, to provide a versatile approach to soft tissue procedures. This ex vivo study investigated the quantity of thermal effects of a dual-wavelength diode laser on porcine lingual mucosa to determine the optimal laser parameters for oral soft tissue biopsies and to improve the reliability of histological evaluation. The presence of thermal damage in the prelesional margins may compromise the diagnostic accuracy, particularly in cases of suspected malignancy. **Methods:** Thirty-six porcine lingual mucosa samples were excised using a diode laser (Wiser 3, Doctor Smile) in continuous wave (CW) and pulsed wave (PW) modes at average powers of 2, 3, and 4 W. The samples, preserved in 5% buffered formalin, underwent histological evaluation to measure epithelial and connective tissue damage. **Results:** The study demonstrated variable thermal effects depending on the laser mode and power settings. Minimal epithelial damage (0.62 mm) was observed at 2 W CW, while maximum damage (3.12 mm) occurred at 4 W pulsed wave (PW). Connective tissue exhibited slightly greater damage than epithelial tissue, with minimal damage (0.53 mm) at 4 W CW and maximum damage (3.19 mm) at 4 W pulsed wave (PW). Statistical analyses were performed using t-tests and ANOVA and revealed significant differences in tissue damage between certain groups, highlighting the impact of laser parameters on thermal effects. **Conclusions:** The dual-wavelength diode laser seems to have good surgical properties and is suitable for managing complex clinical cases. Although the low power average showed minimal thermal damage, for the importance of the diagnosis of suspected lesions of malignancy, a 2 mm prelesional margin should be maintained.

## 1. Introduction

The continuous advancements in dentistry have emphasized the need to investigate the application of lasers in oral surgery due to their numerous advantages, including excellent hemostasis and significant post-operative pain reduction, aiming to achieve minimal invasiveness in biopsy techniques. The main types of lasers used in dentistry include CO_2_ lasers, Er: YAG lasers, Nd: YAG lasers, Er, Cr: YSGG lasers, and diode lasers [1].

The diode laser wavelength (445 nm–1064 nm) is a highly versatile tool with a wide range of applications, including soft tissue surgery, periodontology [2], endodontics [3], tooth whitening, and photo biomodulation (PBM) [4]. The diode laser consists of a solid-state semiconductor made of aluminum, gallium, arsenide, nitrogen and phosphorus, and occasionally indium, which converts electrical energy into light. These wavelengths are selectively absorbed by the primary chromophores of soft tissues, such as protein, collagen, hemoglobin, and melanin.

A dual-wavelength diode laser in dentistry combines two distinct wavelengths, typically 450 nm and 808 nm, to provide a versatile approach to soft tissue procedures. This approach offers advantages over single-wavelength lasers by utilizing the unique properties of each wavelength to achieve optimal coagulation, ablation, and healing.

A flexible optical fiber, typically in the form of a handpiece, transmits the laser beams to the target area. The laser can be used in both continuous and pulsed modes (pulse duration ranging from 0.1 ms to infinity, with programmable frequencies up to 10,000–20,000 Hz). The laser beam is delivered through optical fibers with diameters ranging from 200 to 600 μm [5]. The clinical approach and treatment methods determine the choice between continuous and pulsed modes, contact or non-contact tissue application, and the specific type of tip recommended by the manufacturer [6]. Many diode lasers allow the adjustment of parameters such as power and frequency to minimize tissue damage and enhance precision.

Using dual-wavelength lasers for surgical purposes could achieve hemostatic cutting. In most cases, healing occurs by secondary intention with minimal scar formation, eliminating the need for sutures. Additionally, there is a significant reduction in post-operative pain and inflammation, attributed to cellular biomodulation resulting from residual energy transmission to the tissues during the cutting action [7].

By using both wavelengths, dual-wavelength lasers can achieve a balance between coagulation and ablation, potentially minimizing collateral damage and promoting faster healing.

However, the use of diode lasers for biopsies of soft tissues lesions in the oral cavity remains a topic of debate, particularly regarding the potential thermal damage to the perilesional margins induced by this device: mainly due to the thermal effects generated in the incision area and adjacent tissues, where overheating can lead to histological artifacts of varying severity. The extent of histological artifacts is generally related to the distribution of heat produced by the laser beam within the affected tissue, with evident phenomena of charring and protein denaturation. These alterations compromise the histological evaluation, especially in the case of malignant suspicious lesions undergoing excisional biopsy, leading not only to a possible diagnostic delay but also to potential therapeutic failures. For this reason, although the current literature suggests that a reliable diagnosis can be made using laser techniques, it is essential to understand the true extent of the pathological tissue and define an incision/excision margin that includes a portion of healthy tissue or tissue that has not been excessively compromised by thermal damage. Furthermore, it should be taken into consideration the level of expertise of the surgeon and his manual dexterity in these surgical procedures [8]. The ex vivo study described in this article, resulting from the collaboration between the Department and the “Regina Elena” National Cancer Institute—IFO in Rome, aims to evaluate the thermal effects of a diode laser that uses a combination of two wavelengths on porcine lingual mucosa. The goal is to assess its potential use in oral soft tissue biopsies, define appropriate perilesional resection margins, and ensure accurate histological evaluation, thereby improving diagnostic reliability.

## 2. Materials and Methods

The ex vivo study was conducted on 36 mucosal samples obtained from six tongues of porcine subjects that had died within 24 h of the procedure. This time frame was selected to prevent excessive tissue alteration, which could compromise histological evaluation. Porcine lingual mucosa is considered ideal for such studies due to its histological and physiological characteristics, which closely resemble those of human lingual mucosa.

The appropriate laser safety protocols were employed. The samples were excised using a dual-wavelength diode laser that, in dentistry, combines two distinct wavelengths, 450 nm and 808 nm, specifically, the Wiser 3 [Doctor Smile, Brendola (VI), Italy], in contact mode, employing an initiated surgical tip with a diameter of 300 μm. The fiber was checked during excision for any carbonized build-up to prevent an additional collateral thermal damage, with a length of 5 mm, and operating at a frequency of 10 kHz.

According to the adjusted output power of the laser device, the six experimental groups of the study were as follows, power of 2 W, 3 W, and 4 W in continuous wave (CW) and pulsed wave (PW), and applied, with a total energy of 579.8 J. For 2 W applications, the CW mode resulted in less epithelial and connective damage compared to the PW mode. At 3 W and 4 W, there was no statistically significant difference between CW and PW modes, but the 4 W PW application showed a tendency for higher thermal damage in the connective tissue.

Based on the applied parameters, the 36 samples were divided into six groups, each containing six samples:

- Group A: 2 W in continuous mode (CW).
- Group B: 2 W in pulsed mode (PW).
- Group C: 3 W in continuous mode (CW).
- Group D: 3 W in pulsed mode (PW).
- Group E: 4 W in continuous mode (CW).
- Group F: 4 W in pulsed mode (PW).

The samples were placed in sterile plastic containers filled with 5% buffered formalin to ensure long-term preservation and were subsequently sent to the Pathology Department of the National Cancer Institute “Regina Elena”—IFO in Rome for processing and analysis.

The histological sections obtained, stained with hematoxylin and eosin, were analyzed using an optical microscope at 2.5× magnification. For each sample, two measurements of tissue damage (in mm) were performed using the LAS 4.8 software, assessing epithelial and connective tissue layers in the areas deemed most affected by the laser’s action. To eliminate bias and subjective interpretation, the pathologist conducting the measurements was blinded to the laser application parameters (blind study).

In two of the 36 samples, epithelial damage could not be quantified as the epithelium exhibited severe destruction, leaving unquantifiable fragments. Data analysis was performed using the statistical software SPSS version 27.0.

At first, results were obtained regarding the effects on epithelial and connective tissue, considering both the laser application mode (continuous vs. pulsed) and power levels (2 W, 3 W, 4 W) through independent sample t-tests. Furthermore, an attempt was made to determine whether the laser application mode (CW and PW), regardless of power, could produce different effects on epithelial and connective tissues. Group comparisons in this case were also performed using independent sample *t*-tests.

Finally, to evaluate whether significant differences existed in the mean values of epithelial and connective tissue damage based on laser power (2 W, 3 W, 4 W), disregarding the application mode, a univariate analysis of variance (one-way ANOVA) was performed. Results were considered significant at a *p*-value ≤ 0.10.

## 3. Results

Different thermal effects emerged in the epithelial and connective tissue layers. “Epithelial detachment” was observed in some samples, and it was not possible to evaluate the epithelial damage in two samples because the epithelial layer was destroyed, and these two missing data points do not have a statistically significant impact on the results of the study. Therefore, the overall conclusions drawn from the full set of measurements can still be considered reliable.

**Figure 1:**
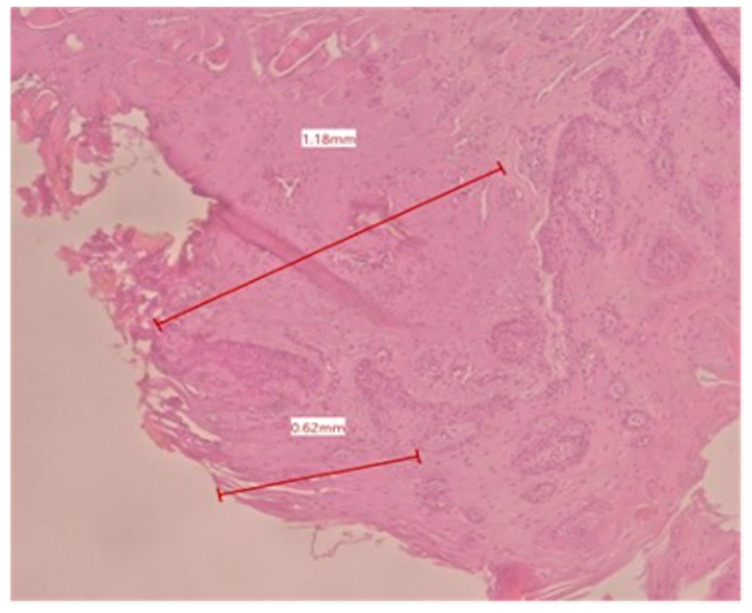
Histological specimen obtained using 2 W in CW shows the epithelial and connective tissue damage with color EE at 2.5× magnification (group A).

**Figure 2:**
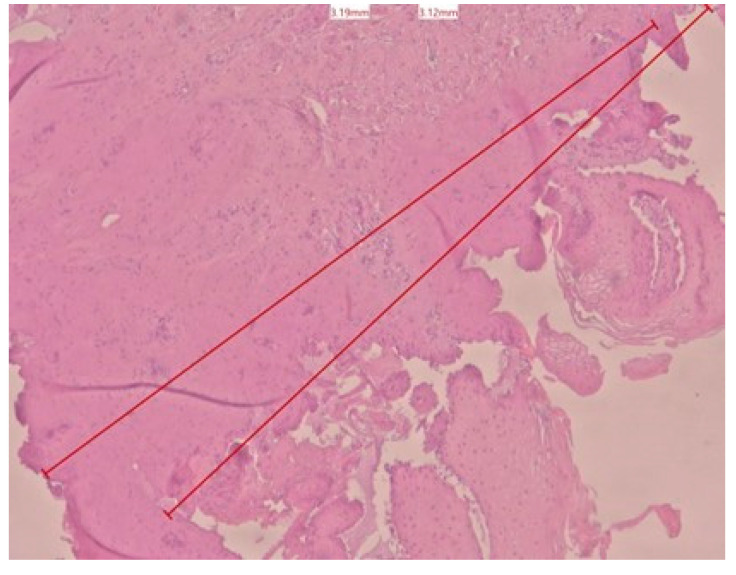
Histological specimen obtained using 4 W in PW epithelial and connective tissue damage with color EE at 2.5× magnification (group F).

**Figure 3:**
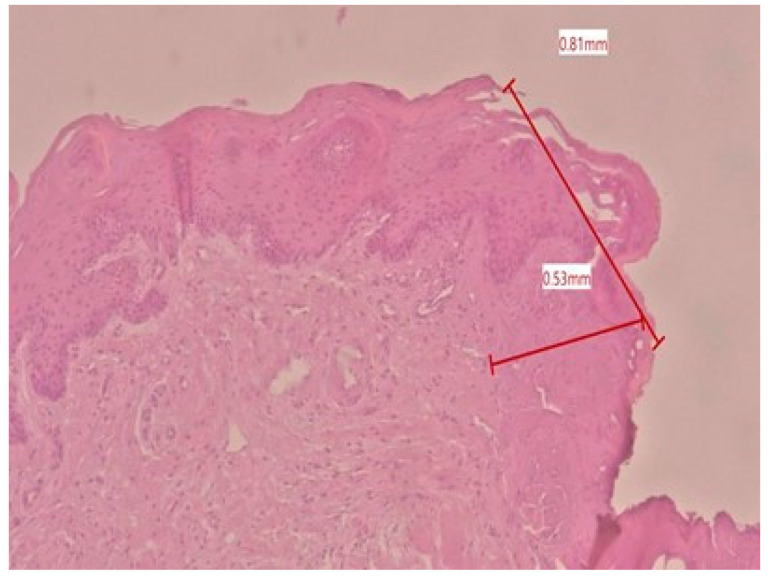
Histological specimen obtained using 4 W epithelial and connective tissue damage in CW with color EE at 2.5× magnification (group E).

In the histological evaluation, the most common laser-induced artifacts emerged in the cells and tissues, like loss of epithelial and sub-epithelial cellular adhesion, epithelial erosion, epithelial spongiosis, carbonization, and vacuolization. Regarding the 70 measurements of tissue damage (34 for the epithelium and 36 for the connective tissue), 10 of these were higher than 2 mm. The minor epithelial damage (0.62 mm, Figure 1) was obtained in a sample taken at 2 W CW, and the major epithelial damage (3.12 mm, Figure 2) was obtained in a sample taken at 4 W PW. In the connective layer, the minor damage (0.53 mm, Figure 3) was obtained in a sample taken at 4 W CW and the major damage (3.19 mm, Figure 1) was obtained at 4 W PW. By comparing individual measurements, the connective tissue damage resulted in being slightly greater than the epithelial damage in 23 out of 36 samples. The average maximum and minimum values of epithelial and connective tissue damages tend to be lower than 2 mm in all six groups (Table 1).

Regarding epithelial damages, average values were compared by *t*-test; the group with greater damages (group B, 2 W, PW) was compared with the others to demonstrate that 2 W in PW mode were the parameters associated with the highest damage in the epithelial tissue (Table 2). Based on data, a statistically significant correlation was obtained (t = 1.94, *p* < 0.01).

On the other hand, no statistically significant correlation was found concerning the comparison between the remaining groups (Table 2). Similarly, comparisons were made between the group associated with the lower average epithelial damage (group A, 2 W CW) and the remaining groups, with statistically significant results in comparison with group B (2 W PW) (t = −1.94, *p* < 0.01) (Table 3). The same analysis was performed on the average connective tissue damage. Group F (4 W PW) seemed to have the highest average value of connective tissue damage, and it was compared with the remaining groups, but the analysis was not statistically significant (t = 0.692, *p* > 0.01) (Table 4). Similarly, comparisons were made between the group associated with the lower average connective tissue damage (group A, 2 W CW) and the remaining groups, without statistically significant results. (t = −1.256, *p* < 0.01) (Table 5). Via t-test, the influence of the application mode of the laser on epithelial and connective tissue was analyzed regardless of the applied powers. Results did not show statistically significant differences among groups. Regarding the epithelial values (t = −1.21, *p* = 0.234), an average damage of 1.22 ± 0.45 was observed in specimens taken at CW and an average damage of 1.45 ± 0.64 in specimens taken at PW. In the connective tissue, an average damage of 1.49 ± 0.46 mm was obtained in specimens taken at CW, and an average damage of 1.65 ± 0.73 mm was obtained in specimens taken at PW (Table 6). The last analysis focused on the epithelial and connective tissue average values obtained at 2 W, 3 W, and 4 W regardless of the application mode of the laser. followed by post hoc comparisons performed with the Bonferroni method. The ANOVA test by post hoc comparisons performed with the Bonferroni method did not show statistically significant differences in the average damages induced by the three powers on the epithelial and connective layers (F(2.33) = 0.271; *p* = 0.765 and (F(2.33) = 0.434; *p* = 0.652, respectively) (Table 7).

## 4. Discussion

Regarding the laser application on the epithelial layer, a correlation between the 2 W CW and higher damages was demonstrated. This result could lead to the hypothesis that the PW, characterized by a lower cutting action than the CW, led to applications of the surgical laser tip in the same mucosal site to obtain its excision; however, this observation cannot be expressed with certainty for CW and PW laser applications at 3 W and 4 W powers, given the absence of significant results. Based on data about the connective tissues, despite the absence of significant results, it could be hypothesized that the lower average damage was obtained by applying the 2 W laser in CW, similarly to the results about epithelial tissue; the higher average damage instead seemed to be associated with the application of the 4 W laser in PW.

This observation could lead to the previous hypothesis elaborated about the epithelium: the repeated application of the surgical laser tip in PW to obtain the excision. The 4 W power could also induce greater damage at the connective level, and since the connective tissue is located deeper than the epithelium, it could be necessary to apply a greater power to obtain the cutting action, consequently also determining greater thermal effects. Moreover, in the case of the comparison between the CW and PW, regardless of the applied powers, despite the lack of statistical evidence, the superiority of the PW application in terms of average damage was possible to note, with reference to the greater connective tissue damages than the epithelial damages.

This consideration could find significance in the wavelengths at which lasers act (445 nm and 808 nm): these have an important affinity for hemoglobin and melanin, the main chromophores richly present in the connective tissue. Consequently, the laser could have a more intense action by penetrating more deeply into the mucosa. Finally, regarding the comparisons between the powers applied regardless of the laser action mode, the lack of significance of the results did not allow for the determination of specific values or power ranges capable of producing greater thermal damage.

However, based on the average values calculated at both the epithelial and connective tissue levels, it could be noted that the damage at the epithelial level is almost similar in most of the samples, while the damage at the connective tissue level is more evident, especially when the laser is applied at 4 W (a parameter that instead at the epithelial level seems to have caused less damage). The explanation for this phenomenon could be in the considerations previously exposed, according to which the laser beam seems to have a more consistent action in the deeper connective layer due to the strong affinity for its biological components.

Laser application in oral biopsies has gained increasing popularity due to its precision, reduction of post-operative pain, faster healing times, and decontamination of the operating site compared to traditional methods [9,10]. Disadvantages include the relatively high cost, a long learning curve depending on the type of laser, thermal damage, the need to modify some operating procedures concerning the cutting capacity of the device, and the need to often use combined wavelengths to obtain the optimal effect in the treatment [11,12].

In an ex vivo study [13], an Er, Cr: YSGG and a dual-wavelength diode laser were employed; nuclei alterations (hyperchromic nuclei, spindle-shaped, and picnotic) were found in each group of both lasers, while in the diode laser groups, the epithelial tissue changes were higher than in the Er, Cr: YSGG laser groups. Cytoplasmic alterations (cell fusion, hyperchromic cytoplasm, and loss of normal cell adhesion) and the loss of intra- and sub-epithelial attachment were higher in the diode laser groups than in the Er, Cr: YSGG laser groups, which had fewer cases of these alterations. Connective tissue changes, including carbonization (thermal necrosis) and vascular alterations (thrombosed, collapsed blood, and lymphatic vessels), were higher in the diode laser groups than in the Er, Cr: YSGG laser groups, but desiccation was observed in all groups. This is related to the photothermal interaction mechanism with different wavelengths, laser radiation power, emission mode, and tissue area irradiated, as seen in the previous studies.

Different in vivo and ex vivo studies have been performed on various types of lasers to evaluate the damage to the tissues produced by the laser heat. Recent studies have reported encouraging results if the laser is used correctly and by a skilled operator; different biopsies can be performed with minimal peripheral thermal damage, which does not affect the proper histological assessment and diagnosis [14].

Furthermore, it is necessary to consider that the evaluation of a laser in an ex vivo study presents important differences compared to an in vivo evaluation. Comparing the results with those of an in vivo study by Palaia et al. [14], in which the histological effects of a 445 nm diode laser were evaluated in clinically benign lesions of 12 patients, the thermal effects at epithelial and connective tissue levels were greater in the in vivo study. The reason for this difference may be the vascularization of the tissues, which was significantly reduced in the mucosa of deceased pigs. This hypothesis can be confirmed by observing the results obtained in the in vivo study in biopsies of pyogenic granuloma, a richly vascularized lesion in which the best thermal effect was obtained, resulting in adequate intraoperative coagulation. At the histological level, greater thermal effects were observed in the connective tissue, compared to the other lesions analyzed, due to the rich amount of hemoglobin, the target chromophore for laser wavelengths between 400 and 500 µm. Therefore, in ex vivo studies, it is important to consider that the results obtained at the histological level cannot fully reflect the results obtained in clinical practice, due to the reduced tissue vascularization, which leads to underestimating the thermal effects of the laser, with repercussions at the histological level.

In general, it is always possible to obtain a reliable diagnosis through laser biopsies, but it is necessary to know the real extension of the pathological tissue and define an incision/excision margin that includes a portion of healthy tissue [15]. Studies conducted about histological in vivo and ex vivo evaluations came to conclude that the diode laser at adequate wavelengths is an important instrument in the case of oral lesions suspected of malignancy, despite a certain amount of perilesional thermal damage [16,17]. In a systematic review by Tenore et al. [18], the thermal effects of a KTP laser (532 nm), a diode laser (445, 808 and 940–980 nm), Nd: YAG (1064 nm), Er:Cr: YSGG (Yttrium, Scandium, Gallium Garnet doped with Erbium and Chromium, 2780 nm), Er: YAG (2940 nm), and a CO_2_ laser were evaluated in oral soft tissue biopsies. A preliminary ex vivo study conducted by Merigo et al. evaluated the thermal effects of different types of lasers (superpulse-CO_2_ laser, Er: YAG laser, diode laser, KTP laser, and Nd: YAG laser) at different levels of power. The best quality of incision was obtained with a 3 W-CO_2_ laser and the 3 W-diode laser statistical result retrieved a significant correlation (*p* < 0.05) for the comparison between these two types of laser, determining a greater increasing in temperature proportional to the depth of the cut in the diode laser than the CO_2_ (differential temperature of 3 W-CO_2_ laser = 22 ± 4.5, differential temperature of 5 W-diode laser = 25 ± 0.6).

In a prospective study by Bargiela-Perez et al., a diode laser (980 nm) was compared with a KTP laser (532 nm) in the resection of oral hyperplastic lesions, obtaining positive results for both types of lasers; a greater superficial absorption capacity emerged from the KTP laser, but the clinical healing aspects were almost the same using one or the other device [19]. The various studies examined recommended the extension of the perilesional margins during the biopsy procedure, considering the use of lasers to be effective, especially in the case of non-suspicious lesions [20], since it is often impossible from a histopathological point of view to determine the extent and degree of dysplasia of the surgical margins. An ex vivo study about a laser application with the same wavelength confirms that the 445 nm laser, such as every laser, creates a minimum thermal effect, which has no implications in the excisions of benign lesions [21].

Peripheral thermal damage may affect the histological assessment of lesion extension; therefore, a comparison is required in each case. For better management of suspected lesions, 1 mm in CW and 0.7 mm in PW should be added in comparison to the scalpel incision [22].

Based on the results of the literature and on the data obtained in this study, which determined a statistically significant correlation between the mean major epithelial damage at 2 W in PW and the mean minor epithelial damage at 2 W CW (1.58 ± 0.51 mm and 1.07 ± 0.30 mm, respectively), it is necessary to evaluate, in each specific case, the possible histological complications, maintaining safety margins of at least 2 mm in continuous and pulsed wave.

## 5. Conclusions

The dual-wavelength diode laser, 450 and 808 nm, could be an excellent alternative for the treatment of benign oral lesions, but it cannot yet be considered the first-choice treatment in dysplastic lesions suspected of malignancy.

However, its use may not be considered an absolute contraindication for any type of lesion since it is extremely important to define perilesional safety margins. The dual-wavelength diode laser evaluated in this study has good surgical properties that make it suitable for managing complex clinical cases, such as vascular lesions and lesions located in areas of difficult access.

On the other hand, it seems to determine thermal effects that are not excessively important in the excision of benign lesions. The diagnostic importance could be determined by maintaining perilesional margins greater than 2 mm, especially in excisional biopsies of lesions suspected of malignancy.

Furthermore, the average power of 2 W CW showed the least thermal damage at 2 mm. It is essential to set the laser parameters according to the operator’s ability and to the individual clinical case, especially when it is necessary to apply different parameters than those recommended by the manufacturer. It is also necessary to consider that the evaluation of a laser in an ex vivo study presents important differences compared to an in vivo evaluation.

Therefore, it is useful to expand the scientific literature on this topic through in vivo studies that can provide information easily comparable to the clinical reality. The lack of significance found in most of the carried-out comparisons suggests carrying out further studies with a larger sample size to confirm or refute the hypotheses and observations presented in this paper.

## Figures and Tables

**Table 1:** Average epithelial and connective tissue damage values for each mode.

Damages	Average Values (mm)
Total epithelial damage	1.33 ± 0.56
Total connective tissue damage	1.57 ± 0.61
Epithelial damage CW	1.22 ± 0.45
Epithelial damage PW	1.45 ± 0.64
Connective tissue damage CW	1.49 ± 0.46
Connective tissue damage PW	1.65 ± 0.73

**Table 2:** Comparison between the group associated with higher average epithelial damage and the remaining groups, with respective (t = −1.94, *p* < 0.01) values.

Group B (2 W PW)	Other Groups	t	*p*
	A (2 W CW)	1.94	0.084
	C (3 W CW)	0.40	0.699
	D (3 W PW)	0.78	0.451
	E (4 W CW)	0.67	0.126
	F (4 W PW)	0.36	0.723

**Table 3:** Comparison between the group associated with lower average epithelial damage and the remaining groups, with respective (t = −1.94, *p* < 0.01) values.

Group A (2 W CW)	Other Groups	t	*p*
	B (2 W PW)	−1.94	0.084
	C (3 W CW)	−1.52	0.162
	D (3 W PW)	−1.23	0.250
	E (4 W CW)	−0.91	0.930
	F (4 W PW)	−0.70	0.504

**Table 4:** Comparison between the groups associated with higher average connective tissue damage and the remaining groups, with respective (t = 0.692, *p* > 0.01) values.

Group F (4W PW)	Other Groups	t	*p*
	A (2 W CW)	0.692	0.516
	B (2 W PW)	0.072	0.945
	C (3 W CW)	0.579	0.581
	D (3 W PW)	0.503	0.634
	E (4 W CW)	0.138	0.856

**Table 5:** Comparison between the groups associated with lower average connective tissue damage and the remaining groups, with respective (t = −1.256, *p* < 0.01) values.

Group A (2W CW)	Other Groups	t	*p*
	B (2 W PW)	−1.256	0.238
	C (3 W CW)	−0.157	0.878
	D (3 W PW)	−0.543	0.599
	E (4 W CW)	−1.010	0.336
	F (4 W PW)	−0.692	0.516

**Table 6:** Comparison between average epithelial and connective tissue damages obtained at CW and PW (t = −1.21, *p* = 0.234) values.

	CW	PW		
	Average Value (mm)	Average Value (mm)	t	*p*
Epithelial damage	1.22 ± 0.45	1.45 ± 0.64	−1.21	0.234
Connective tissue damage	1.49 ± 0.46	1.65 ± 0.73	0.77	0.444

**Table 7:** Comparison between average epithelial and connective tissue damages obtained at 2 W, 3 W, and 4 W (F(2.33) = 0.271; *p* = 0.765 and (F(2.33) = 0.434; *p* = 0.652, respectively).

	2 W	3 W	4 W		
	Average Value (mm)	Average Value (mm)	Average Value (mm)	F(2.33)	*p*
Epithelial damage	1.35 ± 0.48	1.41 ± 0.45	1.21 ± 0.74	0.271	0.765
Connective tissue damage	1.55 ± 0.44	1.46 ± 0.42	1.70 ± 0.88	0.434	0.652

## Data Availability

The original contributions presented in this study are included in the article. Further inquiries can be directed to the corresponding authors.
